# Risks of Adverse Outcomes for Hospitalized COVID-19 Patients during the Four Waves in Brazil According to SARS-CoV-2 Variants, Age Group, and Vaccine Status

**DOI:** 10.3390/v15101997

**Published:** 2023-09-26

**Authors:** Natália Satchiko Hojo-Souza, Waasila Jassat, Daniel Ludovico Guidoni, Fernanda Sumika Hojo de Souza

**Affiliations:** 1 Laboratory of Immunopathology, Oswaldo Cruz Foundation—Minas, Av. Augusto de Lima, 1715, Belo Horizonte 30190-002, MG, Brazil; 2 Division of Public Health Surveillance and Response, National Institute for Communicable Diseases, Johannesburg 2131, South Africa; 3 Department of Computing, Federal University of Ouro Preto, Morro do Cruzeiro Campus, Ouro Preto 35400-000, MG, Brazil

**Keywords:** COVID-19, SARS-CoV-2, variants of concern, waves, adverse outcomes, Brazil

## Abstract

Brazil was hit with four consecutive waves of COVID-19 until 2022 due to the ancestral SARS-CoV-2 (B.1 lineage), followed by the emergence of variants/subvariants. Relative risks of adverse outcomes for COVID-19 patients hospitalized during the four waves were evaluated. Data were extracted from the largest Brazilian database (SIVEP-Gripe), and COVID-19 patients who were hospitalized during the peak of each of the four waves (15-week intervals) were included in this study. The outcomes of in-hospital death, invasive (IMV) and non-invasive (NIV) ventilation requirements, and intensive care unit (ICU) admission were analyzed to estimate the relative risks. A higher risk of in-hospital death was found during the second wave for all age groups, but a significant reduction was observed in the risk of death for the elderly during the third and fourth waves compared to patients in the first wave. There was an increased risk of IMV requirement and ICU admissions during the second wave for patients aged 18–59 years old compared to the first wave. Relative risk analysis showed that booster-vaccinated individuals have lower risks of in-hospital death and IMV requirement in all age groups compared to unvaccinated/partially vaccinated patients, demonstrating the relevance of full/booster vaccination in reducing adverse outcomes for patients who were hospitalized during the variant prevalence.

## 1. Introduction

Throughout the COVID-19 pandemic, there has been a change in the SARS-CoV-2 transmission dynamics, influenced by the number of natural infection cases, level of vaccination adherence, approved vaccines effectiveness, immune escape, and the emergence of new variants [1]. The peaks of the COVID-19 initial waves occurred at different times in distinct countries and regions of a given country, probably due to a combination of factors, such as changes in non-pharmaceutical interventions (NPIs), behavioral diversity, and seasonal factors, in addition to the reduction of transmission by natural infection [1]. In this scenario, the comprehensive vaccination coverage with the primary regimen contributed to reducing cases and mainly hospitalizations and deaths. However, the emergence of the SARS-CoV-2 variants of concern (VOCs), with high transmission advantage, associated with waning immunity over time from natural infection and vaccines, and the decreased effectiveness of current vaccines against circulating VOCs have increased the challenge of controlling the pandemic. In addition to immune escape variants, the relaxation of protective measures, slowness in booster vaccine coverage, and vaccination adherence reduction may increase the risk of new waves.

Since the beginning of the COVID-19 pandemic, Brazil has experienced successive waves characterized by outbreaks due to the ancestral SARS-CoV-2 (lineage B.1), followed by the emergence and predominance of the VOCs [2]. The first wave, which started in March 2020 in Brazil, was caused by the ancestral SARS-CoV-2 reported in Wuhan (China) and mainly affected the elderly with comorbidities [3]. The second wave was caused by the Gamma/P.1 variant (B.1.1.28.1), which originated in the state of Amazonas (Brazil) [4,5]. This highly transmissible, infectious, and virulent variant spread rapidly, with a peak of transmission that occurred in March 2021 in Brazil. It caused high hospitalization rates and deaths, even among young adults [6]. The third wave was driven by the Omicron (B.1.1.529) variant. With numerous mutations in the spike protein, the Omicron variant demonstrated significant immune evasion, capable of causing reinfection and vaccine breakthrough, but vaccinated individuals, particularly with booster doses, have been protected from severe disease and death [7,8]. Despite causing mild/moderate disease and being considered less lethal, the Omicron variant caused a significant increase in daily cases and deaths between January and March 2022 [9], even with more than 80% of the Brazilian population already immunized with their primary vaccine series (two-dose or single-dose) at that time [10]. The fourth COVID-19 wave in Brazil coincides with the prevalence of the Omicron BA.4/BA.5 subvariants, which are more transmissible [11].

As new waves and outbreaks emergence may occur [12], our objective in this study was to characterize the clinical-epidemiological profile and vaccine status by age group of hospitalized Brazilian COVID-19 patients during each of the four pandemic waves. In addition, we determined the relative risks for poor outcomes (death, ICU admission, and ventilation requirement) in the three waves caused by variants/subvariants compared to the first wave, when vaccines were not yet available. Our study can guide public policies for vulnerable subgroups according to the prevalence of the SARS-CoV-2 VOCs.

## 2. Materials and Methods

**Figure 1:**
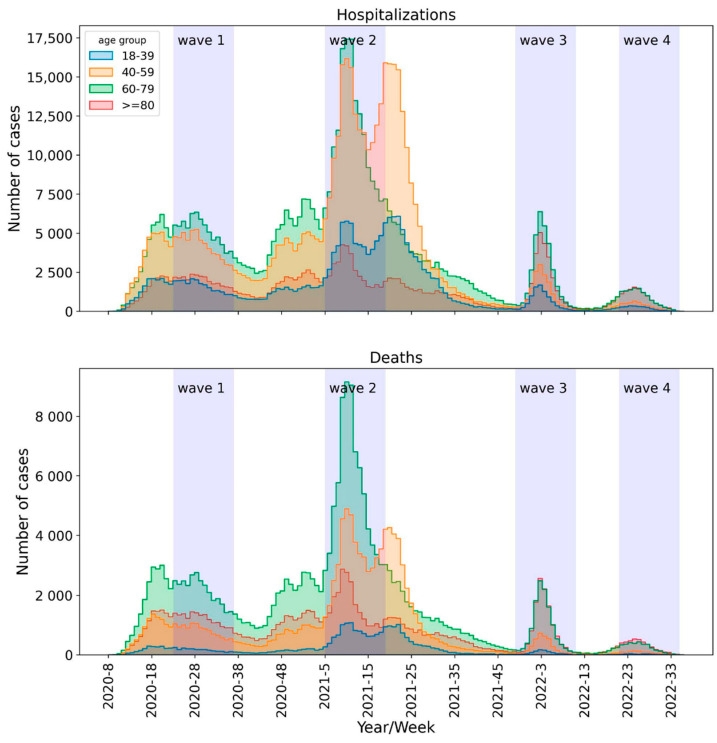
Hospital admissions and deaths of patients with COVID-19 by epidemiological week in Brazil: wave 1 (31 May 2020 to 12 September 2020), wave 2 (31 January 2021 to 15 May 2021), wave 3 (5 December 2021 to 19 March 2022), and wave 4 (22 May 2022 to 3 September 2022).

Data related to four periods of fifteen weeks were retrieved from the SIVEP-Gripe database provided by the Brazilian Ministry of Health. Each period is associated with a COVID-19 wave, with the eighth week of each period characterized by the peak of such a wave. The periods analyzed during the four waves, based on the Brazil Fiocruz genomic surveillance data (https://www.genomahcov.fiocruz.br/dashboard-en/, accessed on 17 August 2023), were defined as follows: wave 1 (31 May 2020 to 12 September 2020), wave 2 (31 January 2021 to 15 May 2021), wave 3 (5 December 2021 to 19 March 2022), and wave 4 (22 May 2022 to 3 September 2022). Figure 1 illustrates the analyzed periods. Data were downloaded from DATASUS on 12 September 2022. Patients hospitalized due to SARS-CoV-2 infection, tested using RT-PCR or antigen, aged ≥ 18 years with available primary outcomes (cure/death) were included. The demographic data, comorbidities, and vaccination status of the patients were retrieved.

Different outcomes were analyzed to estimate the risks during the four waves that hit Brazil: in-hospital deaths, invasive mechanical ventilation (IMV) needs, non-invasive ventilation (NIV) needs, and intensive care unit (ICU) admissions. Descriptive statistics were used to report the features of the studied data: categorical variables were given in absolute numbers and percentages, while continuous variables were reported through medians with the IQR (interquartile range). Poisson regression with a robust variance estimator was used to analyze the association of the different waves and vaccine status with the risk of poor outcomes for hospitalized patients with COVID-19. Stratified by age groups, the adjusted models included the following variables: gender, geographical region, comorbidities, waves, and vaccination status. Patients who had received a booster dose or the complete vaccine schedule (two doses for CoronaVac, Cominarty, and Vaxzevria, or a single dose for Janssen) at least 14 days before symptom onset were considered booster and fully vaccinated, respectively. Patients with inconsistent vaccination data (missing dates or pharmaceutical companies, non-sequential dates for multiple doses, or dates before the start of vaccination) were excluded. Missing data occurred for the comorbidities and some outcomes, including IMV and NIV requirements and ICU admission. Regarding the comorbidities, missing information was filled in as absent for the relative risk analysis. On the other hand, a pairwise deletion was applied to evaluate the relative risks of different poor outcomes as they comprehended the target variable. All analyses were performed using Python (version 3.8.10) and the Scipy statistical package (version 1.7.3). The significance level was set at 0.05.

This retrospective study was based on a publicly available database and did not directly involve patients, not requiring the approval of an ethics committee.

## 3. Results

Brazil has experienced four waves of COVID-19, with 34,754,590 confirmed cases and 687,243 deaths from March 2020 to 19 October 2022 (WHO, 2022). We compared the clinical-epidemiological data, vaccination status, and outcomes from hospitalized patients in the peak period of cases and deaths during the first wave (ancestral SARS-CoV-2), second wave (Gamma/P.1), third wave (Omicron BA.1), and fourth wave (Omicron BA.4/BA.5 subvariants). Without broad genotyping of confirmed SARS-CoV-2 infections, we limited the analysis to data from hospitalized patients around the peak of cases (15 weeks), characterized by substitutions for new variants, to ensure that the cases that were included in this study were due to the circulating variant. We analyzed the relative risks (RRs) for IMV and NIV requirements, ICU admission, and in-hospital death in different age groups in the three waves of variant prevalence compared to the first wave of COVID-19, in addition to full and booster vaccine status compared to unvaccinated and partially vaccinated status.

Data from 193,391 patients were collected in the first wave (W1), while 339,234 patients were compiled in W2, 71,218 in W3, and 29,977 in W4. The median age was 62 years old in W1, and 58 years old, 71 years old, and 74 years old in W2, W3, and W4, respectively. The percentage of males was higher in W1 (55.94%), W2 (55.24%), and W3 (51.22%), while lower in W4 (47.94%) (Table 1). Most hospitalizations occurred in the Southeast region, followed by the South, Northeast, Midwest, and North regions in all the waves. Regarding age groups, we observed an increase in the proportion of ≥60 years old COVID-19 patients who were hospitalized during W3 (70.03%) and W4 (76.27%) compared to W1 (54.90%) and W2 (46.17%). On the other hand, there was a significant reduction in hospitalizations in the 40–59 age group in W3 (19.00%) and W4 (14.84%) compared to the previous waves (Table 1).

Hospitalized patients carrying comorbidities were observed in all waves (Table 2). The most frequent comorbidities in W1 were cardiac disease (41.62%) and diabetes (31.87%). Particularly, in the second wave, a lower prevalence was observed for all comorbidities compared to the first wave (for instance, 36.09% with cardiac disease and 25.80% with diabetes in W2), except for obesity (13.10% in W2 and 8.57% in W1, respectively). On the other hand, the frequencies of hospitalized COVID-19 patients with specific comorbidities during W3 and W4 compared to W1 patients increased significantly: cardiac disease (45.17% and 47.44%, respectively), neurological disease (10.90% and 12.61% vs. 5.76%, respectively), pulmonary disease (8.44% and 10.91% vs. 5.50%, respectively), immunosuppression (6.78% and 7.43% vs. 3.83%, respectively), and kidney disease (8.76% and 8.45% vs. 6.02%, respectively). Different values of n were observed due to missing data. Detailed analysis of symptoms presented by these COVID-19 patients hospitalized during the peak of the four waves was shown in a separate study [13].

The vaccine status of the patients changed significantly along the waves, as the first wave was only composed of unvaccinated patients. In contrast, 7.15% of the patients were partially vaccinated, and 1.01% were fully vaccinated in W2. Moreover, W3 was mainly composed of fully vaccinated patients (50.52%), and W4 presented a high proportion of patients with the booster dose (61.36%) (Table 1). Notably, despite the increase in the hospitalization of elderly patients in W3 and W4, there was a significant decrease in mortality during W4 compared to the previous waves (Table 3), possibly due to the expansion of booster vaccine coverage.

Overall, IMV requirement was markedly lower among patients hospitalized during W4 (15.00%) when more than 60% had received a booster vaccine dose, suggesting that a booster was more effective in protecting against the Omicron variant compared to the full vaccination schedule (50.52% of patients were fully vaccinated in W3) (Table 1 and Table 3). In contrast, IMV requirement was higher among hospitalized patients during the W2 wave (24.32%), characterized by the more pathogenic Gamma/P.1 variant than the others which circulated in Brazil. On the other hand, the proportion of patients requiring NIV were similar in W1, W3, and W4 (~52%), but higher in W2 (61.87%) (Table 3). ICU admissions for COVID-19 patients was similar during W1, W2, and W3 (~38%), showing only a slight reduction in W4 (~33%).

Disaggregated data from hospitalized COVID-19 patients by age group allowed a more detailed analysis of the influence of SARS-CoV-2 variants and vaccination on outcomes throughout the pandemic. During the first wave, when vaccines were unavailable, in-hospital death, IMV requirement, and ICU admission rates were significantly higher in the elderly age groups (60–79 years and ≥80 years). In the second wave, when vaccine coverage was still deficient (8.16%) (Table 1), and there was a prevalence of the most infectious and lethal variant of concern, Gamma/P.1, the rates for in-hospital death and NIV requirement were significantly higher in all age groups compared to the first wave. In the ≥80 years age group, the IMV requirement was slightly lower than in the first wave, possibly due to the preferential vaccination of this group. Notably, during the second wave peak period (W2), there was an increase of more than 75% and more than 92% in the absolute numbers of hospitalized patients and those who died from COVID-19, respectively, compared to the first wave peak period (W1), with deaths having mainly occurred in the age groups of 40–59 years (36,698 deaths vs. 12,353 deaths, respectively) and 60–79 years (61,042 deaths vs. 32,267 deaths, respectively) (Table 4).

**Figure 2:**
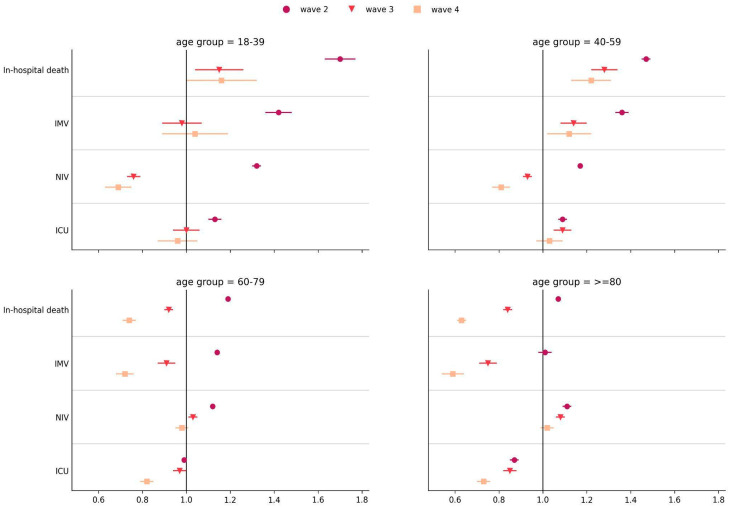
Adjusted relative risks (aRRs) for in-hospital death, IMV requirement, NIV requirement, and ICU admission in COVID-19 patients hospitalized during variant/subvariants predominance relative to patients hospitalized during ancestral lineage/B.1 (wave 1), Gamma/P.1 variant (wave 2), Omicron variant (wave 3), and Omicron BA.4/BA.5 subvariants (wave 4). Error bars represent the 95% confidence interval.

We evaluated the relative risks for in-hospital death, NIV and IMV requirements, and ICU admission according to the variants (waves) and vaccine status in different age groups. Compared to the first wave, W1, there was a significantly higher risk of in-hospital death during W2 for all age groups, being more remarkable among patients in the 18–39 years (aRR = 1.70, 95% CI = [1.63–1.77], *p* < 0.001) and 40–59 years (aRR = 1.47, 95% CI = [1.45–1.50], *p* < 0.001) age groups. On the other hand, during W3 and W4, there was a significant reduction in the risk of in-hospital death in the 60–79 years (aRR = 0.92, 95% CI = [0.90–0.94], *p* < 0.001; aRR = 0.74, 95% CI = [0.71–0.77], *p* < 0.001) and ≥80 years (aRR = 0.84, 95% CI = [0.82–0.86], *p* < 0.001; aRR = 0.63, 95% CI = [0.61–0.66], *p* < 0.001) age groups compared to patients hospitalized in W1. However, in the 18–39 (aRR = 1.15, 95% CI = [1.04–1.26], *p* < 0.005; aRR = 1.16, 95% CI = [1.00–1.34], *p* = 0.055) and 40–59 (aRR = 1.28, 95% CI = [1.22–1.33], *p* < 0.001; aRR = 1.22, 95% CI = [1.13–1.32], *p* < 0.001) age groups, the risk of in-hospital death remained higher than in W1 (Figure 2; Table S1).

Regarding IMV requirement, there was an increased risk during W2, characterized by the Gamma/P.1 variant, for patients in the 18–39 years (aRR = 1.42, 95% CI = [1.36–1.48], *p* < 0.001), 40–59 years (aRR = 1.36, 95% CI = [1.33–1.39], *p* < 0.001) and 60–79 years (aRR = 1.14, 95% CI = [1.13–1.16], *p* < 0.001) age groups compared to W1. In contrast, a significantly reduced risk of IMV requirement by hospitalized patients during W3 and W4 was observed for patients ≥60 years but increased for the 40–59 years (aRR = 1.14, 95% CI = [1.08–1.21], *p* < 0.001; aRR = 1.12, 95% CI = [1.02–1.24], *p* = 0.022) age group, compared to W1 patients (Figure 2; Table S2).

The relative risk for NIV requirement was only lower during W3 and W4 for patients aged 18–39 years (aRR = 0.76, 95% CI = [0.73–0.80], *p* < 0.001; aRR = 0.69, 95% CI = [0.63–0.75], *p* < 0.001) and 40–59 years (aRR = 0.93, 95% CI = [0.91–0.96], *p* < 0.001; aRR = 0.81, 95% CI = [0.77–0.85], *p* < 0.001), who had an increased risk for IMV requirement, as reported (Figure 2; Table S3).

The relative risk for ICU admission during W2 was only increased for patients in the age groups 18–39 years (aRR = 1.13, 95% CI = [1.10–1.16], *p* < 0.001) and 40–59 years (aRR = 1.09, 95% CI = [1.07–1.10], *p* < 0.001) compared to W1. During W3 and W4, the risk for ICU admission was only significantly reduced for patients aged ≥80 years (aRR = 0.85, 95% CI = [0.82–0.88], *p* < 0.001; aRR = 0.73, 95% CI = [0.70–0.76], *p* < 0.001), remaining similar to W1 in the other age groups (Figure 2; Table S4).

**Figure 3:**
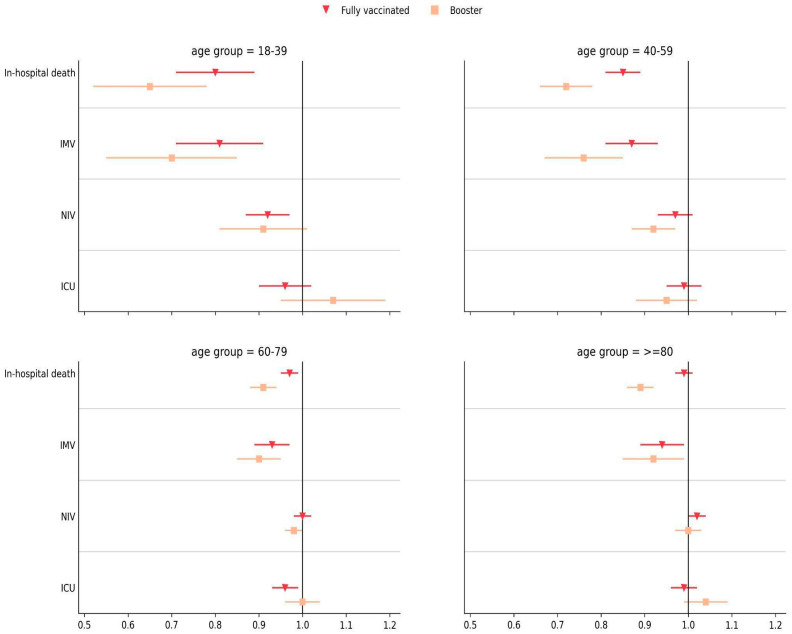
Adjusted relative risks (aRRs) for in-hospital death, IMV requirement, NIV requirement, and ICU admission in COVID-19 patients hospitalized with full and booster vaccination status relative to unvaccinated/partially vaccinated patients in all waves. Error bars represent the 95% confidence interval.

We also evaluated the influence of vaccination (fully and booster) on in-hospital death, ventilation requirement, and ICU admission of hospitalized COVID-19 patients during the prevalence of variants compared to unvaccinated and partially vaccinated patients. Relative risk analysis showed that booster-vaccinated individuals presented lower risks of in-hospital death and IMV requirement in all age groups: 18–39 years (aRR = 0.65, 95% CI = [0.52–0.82], *p* < 0.001; aRR = 0.70, 95% CI = [0.55–0.89], *p* < 0.005), 40–59 (aRR = 0.72, 95% CI = [0.66–0.80], *p* < 0.001; aRR = 0.76, 95% CI = [0.67–0.87], *p* < 0.001), 60–79 (aRR = 0.91, 95% CI = [0.88–0.95], *p* < 0.001; aRR = 0.90, 95% CI = [0.85–0.96], *p* < 0.005), and ≥80 (aRR = 0.89, 95% CI = [0.86–0.92], *p* < 0.001; aRR = 0.92, 95% CI = [0.85–0.99], *p* = 0.032), compared to unvaccinated or partially vaccinated patients (Figure 3; Tables S1 and S2). Similar results were obtained for fully vaccinated patients in all age groups, but with a less prominent risk reduction, as expected. Regarding NIV requirement and ICU admission, relative risks did not show significant reductions between booster/fully vaccinated and unvaccinated/partially vaccinated patients regardless of the age group (Figure 3; Tables S3 and S4).

Considering that ~50% of patients hospitalized during wave W3 were fully vaccinated and ~61% were booster vaccinated in W4 (Table 1), adjusted relative risks of death versus unvaccinated/partially vaccinated patients were calculated for all age groups stratified by waves. Significant relative risk reductions of 17% and 11% for fully vaccinated patients in the 18–39 and 40–59 age groups, respectively, were observed among those hospitalized during W3. Booster-vaccinated patients of all age groups hospitalized during W4 had significant relative risk reductions (23–41%) of death (Table 5).

## 4. Discussion

Brazil has experienced successive well-characterized waves of COVID-19. The first wave occurred before the availability of vaccines. The second wave occurred when this country started their vaccination campaign (starting 17 January 2021), prioritizing more vulnerable groups, such as the elderly, immunosuppressed individuals, and health workers. The third and fourth waves occurred when booster vaccine doses (starting 15 September 2021) [14] were already being administered. The COVID-19 vaccines that have been administered for the Brazilian population in a homologous or heterologous regimen are CoronaVac (Sinovac/Instituto Butantan), Vaxzevria (AstraZeneca/Oxford/Fiocruz), Comirnaty (Pfizer/Wyeth), and Janssen (Johnson & Johnson). These vaccines are based on different platforms and differ regarding their vaccination schedule and degree of efficacy [14].

During the selected periods, a total of 3,817,247 (W1), 6,429,559 (W2), 7,479,019 (W3), and 3,689,290 (W4) cases were nationally confirmed [9], leading to hospitalization rates of 5.06%, 5.28%, 0.95%, and 0.81% from W1 to W4, respectively. Regarding the clinical-epidemiological data, we observed significant differences among hospitalized COVID-19 patients during the four analyzed waves. The increase in the proportion of hospitalized COVID-19 patients aged over 60 years old during W3 and W4 may be due to waning immunity induced by prior vaccination [15,16], as patients in this age group were the first to be vaccinated. Vaccine boosters also prioritized the elderly, which started in September 2021, and it is possible that during W4, such patients could have low antibody titers. The Omicron-mediated variant evasion of vaccine-induced immunity targeting the ancestral SARS-CoV-2 [17,18] may be another contributing factor to the increased hospitalization of the elderly.

The prevalence of comorbidities among hospitalized COVID-19 patients during the four waves also showed significant differences. These data, associated with a higher rate of hospitalization of elderly people during W3 and W4, suggest that comorbidities reflect the age profile of patients. Elderly people are generally more vulnerable due to immunosenescence and are more susceptible to chronic diseases, which can lead to severe COVID-19 [19]. On the other hand, the increased frequency of patients with obesity in W2 may be due to the increased hospitalization of younger obese individuals, who are at a considerably increased risk of severe COVID-19 [20]. Indeed, it was observed that young adults (20–59 years old) had a higher risk of hospitalization and death from COVID-19 during the second wave dominated by the Gamma/P.1 variant [6]. It is important to emphasize that despite missing data for comorbidities and some outcomes, this analysis was conducted to compare the different waves of the pandemic; therefore, we believe that it does not significantly impact the results, since the percentages of missing information remained stable during each of the four periods analyzed.

Our previous study characterized the clinical-epidemiological profile of fully vaccinated individuals who were infected with SARS-CoV-2 (breakthrough infection) during the Gamma/P.1 variant predominance, and we found that the main risk factors for unfavorable outcomes were older age, respiratory compromise, inactivated virus vaccine immunization, and preexisting medical conditions (comorbidities) [21]. The profile of these patients is similar to that of patients who were hospitalized in the subsequent wave dominated by the Omicron variant (W3), suggesting the occurrence of a general profile of patients susceptible to breakthrough infections, regardless of the variant. Notably, the replacement of the Gamma variant with the Delta variant showed that the dissemination was gradual (from June to December 2021) and did not culminate in an increased number of reported cases and deaths, possibly due to immunity acquired via natural infection and/or vaccination before the emergence of the Delta variant in Brazil [22]. Therefore, despite being more transmissible, the Delta variant did not characterize a new wave in Brazil. The third wave was due to the predominance and dissemination of the Omicron variant (B.1.1.529).

Emerging SARS-CoV-2 variants of concern with varying mutations significantly evade antibody immunity induced by COVID-19 vaccines based on the ancestral virus’ spike protein and still make it difficult to avoid disease outbreaks. All monovalent vaccines currently in use target the spike protein from ancestral SARS-CoV-2, which has undergone numerous mutations in the Omicron variant, enabling immune escape. However, studies have shown that an additional dose (third dose) with an mRNA-based vaccine increases protection against the Omicron variant [23,24]. According to our results, the booster vaccine schedule may have protected patients from severe COVID-19 during the emergence of the Omicron subvariants in Brazil.

A study in South Africa showed significantly reduced odds of severe disease among Omicron-infected individuals compared with individuals infected earlier with the Delta variant. However, it was unclear whether the reduced disease severity could be attributed to an intrinsic reduction in Omicron virulence or to immunity acquired from a previous infection, vaccination, or both [25]. Another study also carried out in South Africa showed a reduced risk of in-hospital mortality during the Omicron BA.4/BA.5 wave compared with all previous waves, probably attributed to the combination of lower virulence of the BA.4/BA.5 subvariants, with increased population immunity by expanding vaccine coverage and hybrid immunity (natural infection plus vaccination) [26].

Preliminary observations have indicated that Omicron may spread more rapidly, evade antibodies induced by previous infections or vaccines, and increase mild/moderate breakthrough infection cases [24]. However, severe disease and the need for hospitalization may occur, depending on the scope of primary (fully vaccinated) and booster vaccination coverage, in addition to those unvaccinated. A study carried out with Brazilian COVID-19 patients hospitalized during the predominance of the Omicron variant showed that patients with comorbidities, aged over 60 years, and males had a higher risk of death. At the same time, there was a reduction in the risk of death among those with a booster vaccine dose [27]. Also, vaccination effectively reduced severe COVID-19 and mortality during the fourth wave driven by Omicron in Mexico [28]. In accordance with such studies, booster immunization reduced the risk of IMV requirement and death in W4 in all age groups, demonstrating the importance of the booster dose when no specific vaccine for the Omicron variant/subvariants existed.

Regarding the death aRR for fully vaccinated W3 patients, we only found a risk reduction for patients in the 18–39 and 40–59 age groups. Certain factors, such as natural waning immunity induced by vaccination and greater susceptibility of the elderly due to advanced age and underlying comorbidities, viral variants that escape the immunity induced by the previous non-specific vaccine for this variant [29], could justify this finding. A third dose of an mRNA-based vaccine has been shown to amplify the cross-neutralizing response against the Omicron variant [30]. However, some reports also show that repeated vaccination with the vaccines in use increases the immune response to the ancestral virus and dampens the immune response to the variants [31]. Thus, the outcome could be unfavorable depending on the clinical-epidemiological profile of hospitalized breakthrough infection patients.

The main strength of our study is its large sample size, involving 633,820 hospitalized COVID-19 patients during four different waves of the pandemic that hit Brazil. An important aspect was the characterization of the clinical-demographic profile and vaccine status of hospitalized COVID-19 patients in each of the waves, which allowed us to analyze the relevance of a full and booster vaccination schedule in protecting against adverse outcomes during the prevalence of the Omicron variant of concern and its BA.4/BA.5 subvariants. The inclusion and analysis of data from patients hospitalized during the predominance of the Gamma/P.1 variant also allowed us to comparatively evaluate the poor outcomes caused by this variant, in a period without vaccines against SARS-CoV-2, a result that remains to be reported.

The main limitation of this study is the lack of information on non-hospitalized patients in the database used (SIVEP-Gripe). Empirical data have shown that many individuals with mild or moderate COVID-19 recovered at home, particularly during the predominance of the Omicron variant/subvariants. Another limiting aspect was the non-inclusion of data from hospitalized COVID-19 children and adolescents, as this age group’s vaccination schedule and period significantly differed from adults ≥18 years. Moreover, it is known that registry data might have bias. Considering that the nationwide database is fed by different professionals spread over a large country, there can be differences in the quality of information and missing data. An important point is that the Ministry of Health notification is mandatory, so that the population of interest has national coverage. The forms are completed by healthcare professionals, ensuring better data reliability. Finally, the methodological strategies employed aimed to obtain a precise and robust interpretation of the associations found.

## 5. Conclusions

Our comparative study of four COVID-19 waves in Brazil consistently showed the relevance of full/booster vaccination with vaccines produced against ancestral SARS-CoV-2 in reducing the adverse outcomes for patients that were hospitalized during the prevalence of the variants of concern. These results can guide public policies to prioritize vaccination with booster doses for regions with low vaccination coverage and especially for vulnerable subgroups. Also, these results provide evidence for promoting effective vaccination campaigns, which should be conducted to achieve optimal public health outcomes. Accurate communication strategies can address misconceptions and promote vaccine acceptance. In addition, our study contributes to epidemiological surveillance by characterizing the clinical-epidemiological aspects and vaccine status of patients hospitalized with COVID-19 during the temporal evolution of the pandemic in Brazil, which will allow comparisons with data from other countries. Such data can add new knowledge to emergencies and contribute to adopting preventive and protective interventions for the population, especially for the most vulnerable segments.

## Figures and Tables

**Table 1:** Data from hospitalized COVID-19 patients was stratified by period according to the following predominant variants: W1—ancestral strain/B.1 lineage (31 May 2020 to 12 September 2020), W2—Gamma/P.1 variant (31 January 2021 to 15 May 2021), W3—Omicron variant (5 December 2021 to 19 March 2022), and W4—Omicron BA.4/BA.5 subvariants (22 May 2022 to 3 September 2022).

	W1 (n = 193,391)	W2 (n = 339,234)	W3 (n = 71,218)	W4 (n = 29,977)
**Age, median (IQR), years**	62 (26.00)	58 (21.00)	71 (34.00)	74 (36.00)
**Age group**				
18–39	24,365 (12.60%)	49,312 (14.54%)	7816 (10.97%)	2663 (8.88%)
40–59	62,846 (32.50%)	133,280 (39.29%)	13,528 (19.00%)	4449 (14.84%)
60–79	76,635 (39.63%)	126,891 (37.41%)	28,662 (40.25%)	11,854 (39.54%)
≥80	29,545 (15.28%)	29,751 (8.77%)	21,212 (29.78%)	11,011 (36.73%)
**Gender**				
Female	85,217 (44.06%)	151,835 (44.76%)	34,739 (48.78%)	15,606 (52.06%)
Male	108,174 (55.94%)	187,399 (55.24%)	36,479 (51.22%)	14,371 (47.94%)
**Region**				
Midwest	25,562 (13.22%)	31,530 (9.29%)	5754 (8.08%)	2719 (9.07%)
North	6287 (3.25%)	12,447 (3.67%)	3136 (4.40%)	679 (2.27%)
Northeast	30,260 (15.65%)	41,348 (12.19%)	8656 (12.15%)	2884 (9.62%)
South	31,260 (16.16%)	80,465 (23.72%)	15,450 (21.69%)	6183 (20.63%)
Southeast	100,022 (51.72%)	173,444 (51.13%)	38,222 (53.67%)	17,512 (58.42%)
**Vaccine status**				
Booster	0 (0.00%)	0 (0.00%)	9141 (12.84%)	18,393 (61.36%)
Fully	0 (0.00%)	3432 (1.01%)	35,978 (50.52%)	7518 (25.08%)
Partial	0 (0.00%)	24,248 (7.15%)	3580 (5.03%)	1049 (3.50%)
Unvaccinated	193,391 (100.00%)	311,554 (91.84%)	22,519 (31.62%)	3017 (10.06%)

**Table 2:** Comorbidities’ frequency in hospitalized COVID-19 patients was stratified by period according to the following predominant variants: W1—ancestral lineage/B.1 (31 May 2020 to 12 September 2020), W2–Gamma/P.1 variant (31 January 2021 to 15 May 2021), W3—Omicron variant (5 December 2021 to 19 March 2022), and W4—Omicron BA.4/BA.5 subvariants (22 May 2022 to 3 September 2022).

	W1 (n = 193,391)	W2 (n = 339,234)	W3 (n = 71,218)	W4 (n = 29,977)
Cardiac disease	n = 171,767	n = 300,788	n = 60,415	n = 25,555
	71,486 (41.62%)	108,552 (36.09%)	27,292 (45.17%)	12,123 (47.44%)
Hematological disease	n = 147,698	n = 261,765	n = 50,213	n = 21,338
	1567 (1.06%)	1706 (0.65%)	813 (1.62%)	393 (1.84%)
Down’s syndrome	n = 147,701	n = 261,604	n = 50,089	n = 21,306
	441 (0.30%)	828 (0.32%)	242 (0.48%)	87 (0.41%)
Liver disease	n = 147,575	n = 261,454	n = 50,097	n = 21,331
	1933 (1.31%)	2159 (0.83%)	861 (1.72%)	395 (1.85%)
Asthma	n = 148,643	n = 263,535	n = 50,313	n = 21,498
	5339 (3.59%)	8038 (3.05%)	1725 (3.43%)	867 (4.03%)
Diabetes	n = 165,434	n = 289,125	n = 57,070	n = 23,936
	52,729 (31.87%)	74,602 (25.80%)	18,233 (31.95%)	7275 (30.39%)
Neurologic disease	n = 149,868	n = 263,795	n = 52,003	n = 22,161
	8625 (5.76%)	8818 (3.34%)	5668 (10.90%)	2795 (12.61%)
Pulmonary disease	n = 149,696	n = 264,003	n = 51,517	n = 22,008
	8239 (5.50%)	8583 (3.25%)	4349 (8.44%)	2401 (10.91%)
Immunosuppression	n = 148,417	n = 262,564	n = 51,055	n = 21,699
	5678 (3.83%)	5723 (2.18%)	3464 (6.78%)	1612 (7.43%)
Obesity	n = 148,911	n = 271,813	n = 50,854	n = 21,341
	12,762 (8.57%)	35,609 (13.10%)	4320 (8.49%)	1253 (5.87%)
Kidney disease	n = 149,768	n = 263,708	n = 51,484	n = 21,828
	9009 (6.02%)	9071 (3.44%)	4510 (8.76%)	1845 (8.45%)
Other comorbidity	n = 163,410	n = 289,066	n = 59,418	n = 25,120
	57,513 (35.20%)	87,598 (30.30%)	25,366 (42.69%)	11,337 (45.13%)

**Table 3:** Outcomes in hospitalized COVID-19 patients was stratified by period according to the following predominant variants: W1—ancestral lineage/B.1 (31 May 2020 to 12 September 2020), W2—Gamma/P.1 variant (31 January 2021 to 15 May 2021), W3—Omicron variant (5 December 2021 to 19 March 2022), and W4—Omicron BA.4/BA.5 subvariants (22 May 2022 to 3 September 2022).

	W1 (n = 193,391)	W2 (n = 339,234)	W3 (n = 71,218)	W4 (n = 29,977)
In-hospital death	n = 193,391	n = 339,234	n = 71,218	n = 29,977
	65,148 (33.69%)	125,231 (36.92%)	25,634 (35.99%)	8527 (28.45%)
IMV	n = 171,605	n = 307,010	n = 63,026	n = 26,440
	36,457 (21.24%)	74,676 (24.32%)	12,056 (19.13%)	3966 (15.00%)
NIV	n = 171,605	n = 307,010	n = 63,026	n = 26,440
	91,059 (53.06%)	189,946 (61.87%)	33,769 (53.58%)	13,521 (51.14%)
ICU	n = 178,350	n = 312,958	n = 65,604	n = 27,699
	69,287 (38.85%)	118,250 (37.78%)	24,873 (37.91%)	9253 (33.41%)

**Table 4:** Outcomes’ frequency in hospitalized COVID-19 patients was stratified by age group and period according to the following predominant variants: W1—ancestral lineage/B.1 (31 May 2020 to 12 September 2020), W2—Gamma/P.1 variant (31 January 2021 to 15 May 2021), W3—Omicron variant (5 December 2021 to 19 March 2022), and W4—Omicron BA.4/BA.5 subvariants (22 May 2022 to 3 September 2022).

	W1 (n = 193,391)	W2 (n = 339,234)	W3 (n = 71,218)	W4 (n = 29,977)
In-hospital death	n = 193,391	n = 339,234	n = 71,218	n = 29,977
18–39	10.22% (2490/24,365)	16.78% (8275/49,312)	10.85% (848/7816)	9.65% (257/2663)
40–59	19.66% (12,353/62,846)	27.53% (36,698/133,280)	24.64% (3333/13,528)	21.06% (937/4449)
60–79	42.10% (32,267/76,635)	48.11% (61,042/126,891)	38.17% (10,941/28,662)	29.26% (3468/11,854)
≥80	61.05% (18,038/29,545)	64.59% (19,216/29,751)	49.56% (10,512/21,212)	35.10% (3865/11,011)
IMV	n = 171,605	n = 307,010	n = 63,026	n = 26,440
18–39	11.80% (2561/21,696)	16.86% (7554/44,798)	10.63% (721/6781)	10.03% (228/2274)
40–59	16.50% (9227/55,918)	22.06% (26,657/120,840)	18.57% (2218/11,946)	16.25% (626/3852)
60–79	26.42% (17,959/67,964)	29.46% (33,782/114,689)	22.88% (5825/25,463)	17.37% (1819/10,474)
≥80	25.78% (6710/26,027)	25.05% (6683/26,683)	17.48% (3292/18,836)	13.14% (1293/9840)
NIV	n = 171,605	n = 307,010	n = 63,026	n = 26,440
18–39	47.14% (10,227/21,696)	63.16% (28,294/44,798)	33.30% (2258/6781)	29.38% (668/2274)
40–59	54.14% (30,274/55,918)	63.45% (76,673/120,840)	48.63% (5809/11,946)	41.07% (1582/3852)
60–79	52.65% (35,785/67,964)	59.29% (67,995/114,689)	54.46% (13,867/25,463)	51.87% (5433/10,474)
≥80	56.76% (14,773/26,027)	63.65% (16,984/26,683)	62.83% (11,835/18,836)	59.33% (5838/9840)
ICU	n = 178,350	n = 312,958	n = 65,604	n = 27,699
18–39	27.38% (6197/22,633)	31.04% (14,172/45,656)	26.17% (1880/7183)	25.80% (626/2426)
40–59	33.76% (19,664/58,253)	35.94% (44,275/123,201)	37.52% (4681/12,476)	34.29% (1402/4089)
60–79	44.08% (31,066/70,482)	42.31% (49,435/116,829)	41.55% (11,000/26,472)	35.51% (3895/10,968)
≥80	45.81% (12,360/26,982)	38.02% (10,368/27,272)	37.55% (7312/19,473)	32.60% (3330/10,216)

**Table 5:** Adjusted relative risks for death of fully vaccinated versus unvaccinated/partially vaccinated patients hospitalized during W3, and booster-vaccinated versus unvaccinated/partially vaccinated patients hospitalized during W4.

Age Group	Fully Vaccinated × Unvaccinated/Partially Vaccinated (W3)	Booster Vaccinated × Unvaccinated/Partially Vaccinated (W4)
18–39	0.83 (0.73–0.94), *p* < 0.005	0.73 (0.54–0.98), *p* = 0.034
40–59	0.89 (0.84–0.95), *p* < 0.001	0.59 (0.52–0.68), *p* < 0.001
60–79	0.97 (0.94–1.00), *p* = 0.057	0.77 (0.71–0.83), *p* < 0.001
≥80	1.00 (0.97–1.03), *p* = 0.796	0.70 (0.65–0.75), *p* < 0.001

## Data Availability

The data used in this study are publicly available at: opendatasus.saude.gov.br (accessed on 12 September 2022).

## Supplementary Materials

The following supporting information can be downloaded at: https://www.mdpi.com/article/10.3390/v15101997/s1, Table S1: Adjusted relative risks (aRRs) for in-hospital death in COVID-19 patients hospitalized during variant/subvariants predominance relative to patients hospitalized during ancestral lineage/B.1 (wave 1), Gamma/P.1 variant (wave 2), Omicron variant (wave 3), and Omicron BA.4/BA.5 subvariants (wave 4), stratified by age. Table S2: Adjusted relative risks (aRRs) for invasive mechanical ventilation (IMV) need in COVID-19 patients hospitalized during variant/subvariants predominance relative to patients hospitalized during ancestral lineage/B.1 (wave 1), Gamma/P.1 variant (wave 2), Omicron variant (wave 3), and Omicron BA.4/BA.5 subvariants (wave 4), stratified by age. Table S3: Adjusted relative risks (aRRs) for non-invasive ventilation (NIV) need in COVID-19 patients hospitalized during variant/subvariants predominance relative to patients hospitalized during ancestral lineage/B.1 (wave 1), Gamma/P.1 variant (wave 2), Omicron variant (wave 3), and Omicron BA.4/BA.5 subvariants (wave 4), stratified by age. Table S4: Adjusted relative risks (aRRs) for intensive care unit (ICU) admission in COVID-19 patients hospitalized during variant/subvariants predominance relative to patients hospitalized during ancestral lineage/B.1 (wave 1), Gamma/P.1 variant (wave 2), Omicron variant (wave 3), and Omicron BA.4/BA.5 subvariants (wave 4), stratified by age.
