# Supplementary material for: Risks of Adverse Outcomes for Hospitalized COVID-19 Patients during the Four Waves in Brazil According to SARS-CoV-2 Variants, Age Group, and Vaccine Status
Source: Viruses. 2023 Sep 26;15(10):1997. doi: 10.3390/v15101997 (PMC10610718; doi:10.3390/v15101997)
Supplement: Supplementary file 1 [file viruses-15-01997-s001.zip › viruses-2590740-supplementary.pdf]

Table S1. Adjusted relative risks (aRR) for **in-hospital death** in COVID-19 patients hospitalized during variant/subvariants predominance relative to patients hospitalized during Ancestral lineage/B.1 (wave 1), Gamma/P.1 variant (wave 2), Omicron variant (wave 3) and Omicron BA.4/BA.5 subvariants (wave 4), stratified by age.

**Age group**	**18-39**			**40-59**			**60-79**			**>=80**		
**Covariate**	**aRR**	**CI 95%**	**p value**	**aRR**	**CI 95%**	**p value**	**aRR**	**CI 95%**	**p value**	**aRR**	**CI 95%**	**p value**
**Male**	1.09	(1.05-1.13)	<0.001	1.07	(1.06-1.09)	<0.001	1.13	(1.12-1.14)	<0.001	1.11	(1.10-1.13)	<0.001
**Cardiac disease**	1.29	(1.22-1.35)	<0.001	1.19	(1.17-1.21)	<0.001	1.07	(1.06-1.08)	<0.001	1.01	(1.00-1.02)	0.059
**Hematological disease**	1.19	(0.95-1.49)	0.128	1.05	(0.96-1.15)	0.283	1.11	(1.07-1.16)	<0.001	1.05	(0.99-1.11)	0.124
**Down's syndrome**	1.89	(1.61-2.21)	<0.001	1.37	(1.20-1.55)	<0.001	0.88	(0.78-0.99)	0.027	0.84	(0.72-1.00)	0.044
**Liver disease**	1.15	(0.89-1.49)	0.297	1.53	(1.45-1.62)	<0.001	1.26	(1.22-1.31)	<0.001	1.14	(1.08-1.21)	<0.001
**Asthma**	0.92	(0.83-1.01)	0.076	0.94	(0.89-0.98)	0.007	0.90	(0.88-0.93)	<0.001	0.91	(0.87-0.95)	<0.001
**Diabetes**	1.44	(1.36-1.52)	<0.001	1.28	(1.26-1.30)	<0.001	1.10	(1.09-1.11)	<0.001	1.02	(1.01-1.03)	<0.005
**Neurological disease**	1.69	(1.52-1.88)	<0.001	1.42	(1.36-1.49)	<0.001	1.26	(1.24-1.29)	<0.001	1.14	(1.13-1.16)	<0.001
**Pulmonary disease**	1.05	(0.88-1.27)	0.579	1.35	(1.29-1.42)	<0.001	1.23	(1.21-1.25)	<0.001	1.13	(1.11-1.15)	<0.001

**Immunosuppression**	2.23	(2.06-2.41)	<0.001	1.60	(1.54-1.66)	<0.001	1.27	(1.24-1.30)	<0.001	1.11	(1.07-1.14)	<0.001
**Kidney disease**	1.61	(1.46-1.78)	<0.001	1.65	(1.60-1.70)	<0.001	1.31	(1.29-1.33)	<0.001	1.18	(1.15-1.20)	<0.001
**Obesity**	1.99	(1.92-2.07)	<0.001	1.47	(1.44-1.50)	<0.001	1.18	(1.17-1.20)	<0.001	1.07	(1.04-1.10)	<0.001
**Other comorbidity**	1.58	(1.53-1.65)	<0.001	1.32	(1.30-1.34)	<0.001	1.13	(1.12-1.14)	<0.001	1.07	(1.06-1.08)	<0.001
**Fully vaccinated**	0.80	(0.71-0.90)	<0.001	0.85	(0.81-0.90)	<0.001	0.97	(0.95-1.00)	0.035	0.99	(0.97-1.01)	0.332
**Booster**	0.65	(0.52-0.82)	<0.001	0.72	(0.66-0.80)	<0.001	0.91	(0.88-0.95)	<0.001	0.89	(0.86-0.92)	<0.001
**Wave 2**	1.70	(1.63-1.77)	<0.001	1.47	(1.45-1.50)	<0.001	1.19	(1.18-1.20)	<0.001	1.07	(1.06-1.09)	<0.001
**Wave 3**	1.15	(1.04-1.26)	<0.005	1.28	(1.22-1.33)	<0.001	0.92	(0.90-0.94)	<0.001	0.84	(0.82-0.86)	<0.001
**Wave 4**	1.16	(1.00-1.34)	0.055	1.22	(1.13-1.32)	<0.001	0.74	(0.71-0.77)	<0.001	0.63	(0.61-0.66)	<0.001
**South**	0.90	(0.86-0.94)	<0.001	0.97	(0.95-0.98)	<0.001	0.99	(0.98-1.01)	0.338	1.01	(0.99-1.03)	0.216
**North**	1.51	(1.41-1.62)	<0.001	1.45	(1.40-1.50)	<0.001	1.29	(1.26-1.32)	<0.001	1.14	(1.11-1.17)	<0.001
**Northeast**	1.42	(1.36-1.49)	<0.001	1.28	(1.26-1.31)	<0.001	1.17	(1.16-1.19)	<0.001	1.12	(1.10-1.14)	<0.001
**Midwest**	1.12	(1.06-1.18)	<0.001	1.08	(1.06-1.11)	<0.001	1.04	(1.03-1.06)	<0.001	1.01	(0.99-1.03)	0.467

Table S2. Adjusted relative risks (aRR) for **invasive mechanical ventilation (IMV)** need in COVID-19 patients hospitalized during variant/subvariants predominance relative to patients hospitalized during Ancestral lineage/B.1 (wave 1), Gamma/P.1 variant (wave 2), Omicron variant (wave 3) and Omicron BA.4/BA.5 subvariants (wave 4), stratified by age.

**Age group**	**18-39**			**40-59**			**60-79**			**>=80**		
**Covariate**	**aRR**	**CI 95%**	**p value**	**aRR**	**CI 95%**	**p value**	**aRR**	**CI 95%**	**p value**	**aRR**	**CI 95%**	**p value**
**Male**	1.06	(1.03-1.10)	<0.005	1.06	(1.04-1.08)	<0.001	1.10	(1.08-1.12)	<0.001	1.21	(1.18-1.24)	<0.001
**Cardiac disease**	1.20	(1.14-1.27)	<0.001	1.18	(1.16-1.20)	<0.001	1.11	(1.10-1.13)	<0.001	1.11	(1.08-1.14)	<0.001
**Hematological disease**	1.04	(0.83-1.31)	0.749	1.06	(0.95-1.18)	0.326	1.09	(1.01-1.17)	0.020	0.96	(0.83-1.10)	0.536
**Down's syndrome**	1.85	(1.59-2.16)	<0.001	1.40	(1.22-1.60)	<0.001	0.85	(0.71-1.01)	0.062	0.95	(0.69-1.30)	0.734
**Liver disease**	1.15	(0.90-1.48)	0.262	1.36	(1.26-1.47)	<0.001	1.19	(1.13-1.26)	<0.001	1.24	(1.08-1.41)	<0.005
**Asthma**	1.00	(0.92-1.10)	0.953	1.01	(0.96-1.07)	0.620	0.96	(0.91-1.00)	0.049	0.97	(0.89-1.06)	0.499
**Diabetes**	1.37	(1.29-1.45)	<0.001	1.21	(1.18-1.23)	<0.001	1.08	(1.07-1.10)	<0.001	1.08	(1.05-1.11)	<0.001
**Neurological disease**	1.72	(1.55-1.91)	<0.001	1.36	(1.28-1.43)	<0.001	1.08	(1.05-1.12)	<0.001	0.89	(0.86-0.93)	<0.001
**Pulmonary disease**	1.08	(0.91-1.28)	0.383	1.24	(1.17-1.32)	<0.001	1.26	(1.22-1.29)	<0.001	1.15	(1.10-1.21)	<0.001

**Immunosuppression**	1.55	(1.41-1.70)	<0.001	1.27	(1.21-1.34)	<0.001	1.08	(1.04-1.12)	<0.001	1.00	(0.92-1.08)	0.968
**Kidney disease**	1.53	(1.38-1.70)	<0.001	1.44	(1.39-1.51)	<0.001	1.25	(1.22-1.28)	<0.001	1.20	(1.15-1.26)	<0.001
**Obesity**	2.00	(1.93-2.08)	<0.001	1.69	(1.65-1.72)	<0.001	1.44	(1.41-1.47)	<0.001	1.38	(1.30-1.46)	<0.001
**Other comorbidity**	1.43	(1.37-1.49)	<0.001	1.26	(1.24-1.29)	<0.001	1.15	(1.13-1.17)	<0.001	1.08	(1.05-1.11)	<0.001
**Fully vaccinated**	0.81	(0.71-0.91)	<0.005	0.87	(0.81-0.93)	<0.001	0.93	(0.89-0.96)	<0.001	0.94	(0.89-0.99)	0.015
**Booster**	0.70	(0.55-0.89)	<0.005	0.76	(0.67-0.87)	<0.001	0.90	(0.85-0.96)	<0.005	0.92	(0.85-0.99)	0.032
**Wave 2**	1.42	(1.36-1.48)	<0.001	1.36	(1.33-1.39)	<0.001	1.14	(1.13-1.16)	<0.001	1.01	(0.98-1.04)	0.519
**Wave 3**	0.98	(0.89-1.08)	0.702	1.14	(1.08-1.21)	<0.001	0.91	(0.87-0.94)	<0.001	0.75	(0.71-0.78)	<0.001
**Wave 4**	1.04	(0.89-1.22)	0.614	1.12	(1.02-1.24)	0.022	0.72	(0.68-0.77)	<0.001	0.59	(0.54-0.64)	<0.001
**South**	1.19	(1.14-1.25)	<0.001	1.25	(1.23-1.28)	<0.001	1.23	(1.20-1.25)	<0.001	1.07	(1.03-1.11)	<0.001
**North**	1.37	(1.26-1.49)	<0.001	1.44	(1.38-1.51)	<0.001	1.40	(1.35-1.45)	<0.001	1.58	(1.48-1.68)	<0.001
**Northeast**	1.42	(1.36-1.50)	<0.001	1.38	(1.34-1.42)	<0.001	1.43	(1.40-1.46)	<0.001	1.58	(1.53-1.63)	<0.001
**Midwest**	1.22	(1.16-1.29)	<0.001	1.22	(1.18-1.25)	<0.001	1.27	(1.24-1.30)	<0.001	1.47	(1.41-1.54)	<0.001

Table S3. Adjusted relative risks (aRR) for **non-invasive ventilation (NIV)** need in COVID-19 patients hospitalized during variant/subvariants predominance relative to patients hospitalized during Ancestral lineage/B.1 (wave 1), Gamma/P.1 variant (wave 2), Omicron variant (wave 3) and Omicron BA.4/BA.5 subvariants (wave 4), stratified by age.

**Age group**	**18-39**			**40-59**			**60-79**			**>=80**		
**Covariate**	**aRR**	**CI 95%**	**p value**	**aRR**	**CI 95%**	**p value**	**aRR**	**CI 95%**	**p value**	**aRR**	**CI 95%**	**p value**
**Male**	1.15	(1.14-1.17)	<0.001	1.01	(1.01-1.02)	<0.005	0.96	(0.95-0.97)	<0.001	0.94	(0.93-0.95)	<0.001
**Cardiac disease**	1.05	(1.03-1.07)	<0.001	1.03	(1.02-1.03)	<0.001	1.01	(1.00-1.01)	0.081	1.02	(1.01-1.03)	<0.005
**Hematological disease**	0.94	(0.85-1.04)	0.250	0.91	(0.86-0.97)	<0.005	0.96	(0.92-1.00)	0.053	1.00	(0.95-1.07)	0.904
**Down's syndrome**	0.91	(0.83-1.00)	0.060	0.93	(0.86-1.01)	0.097	1.03	(0.94-1.13)	0.497	0.84	(0.70-1.01)	0.057
**Liver disease**	0.88	(0.78-1.00)	0.042	0.83	(0.79-0.87)	<0.001	0.90	(0.86-0.93)	<0.001	0.89	(0.83-0.96)	<0.005
**Asthma**	1.05	(1.02-1.09)	<0.005	1.03	(1.01-1.06)	<0.005	1.03	(1.00-1.05)	0.030	1.02	(0.99-1.06)	0.225
**Diabetes**	1.01	(0.98-1.04)	0.557	0.99	(0.98-1.00)	0.233	1.00	(0.99-1.00)	0.300	0.99	(0.98-1.00)	0.118
**Neurological disease**	1.01	(0.96-1.07)	0.639	0.96	(0.93-1.00)	0.026	1.02	(1.00-1.03)	0.080	1.08	(1.07-1.10)	<0.001
**Pulmonary disease**	1.14	(1.07-1.22)	<0.001	1.04	(1.00-1.07)	0.025	1.01	(1.00-1.03)	0.174	1.04	(1.02-1.06)	<0.001

**Immunosuppression**	0.92	(0.87-0.97)	<0.005	0.92	(0.89-0.94)	<0.001	0.94	(0.92-0.97)	<0.001	0.99	(0.96-1.03)	0.583
**Kidney disease**	0.87	(0.82-0.93)	<0.001	0.87	(0.84-0.89)	<0.001	0.88	(0.86-0.90)	<0.001	0.93	(0.91-0.95)	<0.001
**Obesity**	1.00	(0.98-1.02)	0.970	0.90	(0.89-0.91)	<0.001	0.88	(0.87-0.89)	<0.001	0.92	(0.89-0.95)	<0.001
**Other comorbidity**	0.93	(0.92-0.95)	<0.001	0.96	(0.95-0.97)	<0.001	0.97	(0.96-0.98)	<0.001	1.02	(1.01-1.03)	<0.005
**Fully vaccinated**	0.92	(0.87-0.97)	0.005	0.97	(0.93-1.00)	0.046	1.00	(0.98-1.03)	0.645	1.02	(1.00-1.04)	0.047
**Booster**	0.91	(0.81-1.02)	0.105	0.92	(0.87-0.98)	0.008	0.98	(0.96-1.01)	0.211	1.00	(0.97-1.02)	0.750
**Wave 2**	1.32	(1.30-1.34)	<0.001	1.17	(1.16-1.18)	<0.001	1.12	(1.11-1.13)	<0.001	1.11	(1.09-1.12)	<0.001
**Wave 3**	0.76	(0.73-0.80)	<0.001	0.93	(0.91-0.96)	<0.001	1.03	(1.01-1.05)	<0.005	1.08	(1.06-1.10)	<0.001
**Wave 4**	0.69	(0.63-0.75)	<0.001	0.81	(0.77-0.85)	<0.001	0.98	(0.95-1.01)	0.225	1.02	(0.99-1.04)	0.258
**South**	0.99	(0.97-1.00)	0.084	0.96	(0.95-0.97)	<0.001	0.93	(0.92-0.94)	<0.001	1.00	(0.99-1.02)	0.621
**North**	0.92	(0.89-0.95)	<0.001	0.88	(0.86-0.90)	<0.001	0.81	(0.79-0.83)	<0.001	0.82	(0.79-0.85)	<0.001
**Northeast**	0.89	(0.87-0.91)	<0.001	0.91	(0.90-0.92)	<0.001	0.84	(0.83-0.85)	<0.001	0.86	(0.84-0.87)	<0.001
**Midwest**	0.92	(0.90-0.94)	<0.001	0.90	(0.89-0.91)	<0.001	0.85	(0.84-0.86)	<0.001	0.81	(0.79-0.83)	<0.001

Table S4. Adjusted relative risks (aRR) for **intensive care unit (ICU)** admission in COVID-19 patients hospitalized during variant/subvariants predominance relative to patients hospitalized during Ancestral lineage/B.1 (wave 1), Gamma/P.1 variant (wave 2), Omicron variant (wave 3) and Omicron BA.4/BA.5 subvariants (wave 4), stratified by age.

**Age group**	**18-39**			**40-59**			**60-79**			**>=80**		
**Covariate**	**aRR**	**CI 95%**	**p value**	**aRR**	**CI 95%**	**p value**	**aRR**	**CI 95%**	**p value**	**aRR**	**CI 95%**	**p value**
**Male**	1.11	(1.08-1.13)	<0.001	1.12	(1.11-1.13)	<0.001	1.10	(1.09-1.11)	<0.001	1.12	(1.11-1.14)	<0.001
**Cardiac disease**	1.17	(1.13-1.21)	<0.001	1.15	(1.14-1.17)	<0.001	1.12	(1.11-1.13)	<0.001	1.12	(1.10-1.14)	<0.001
**Hematological disease**	1.00	(0.87-1.14)	0.954	1.07	(1.00-1.15)	0.062	1.11	(1.06-1.17)	<0.001	1.01	(0.93-1.11)	0.796
**Down's syndrome**	1.44	(1.30-1.60)	<0.001	1.20	(1.09-1.32)	<0.001	1.00	(0.89-1.12)	0.989	0.77	(0.60-0.99)	0.045
**Liver disease**	1.09	(0.94-1.27)	0.249	1.14	(1.09-1.21)	<0.001	1.07	(1.03-1.12)	<0.005	1.08	(0.99-1.18)	0.078
**Asthma**	1.10	(1.04-1.15)	<0.005	1.02	(0.98-1.06)	0.293	0.96	(0.93-0.99)	0.007	1.04	(0.98-1.10)	0.188
**Diabetes**	1.26	(1.22-1.31)	<0.001	1.13	(1.11-1.14)	<0.001	1.05	(1.04-1.06)	<0.001	1.07	(1.05-1.09)	<0.001
**Neurological disease**	1.35	(1.26-1.44)	<0.001	1.20	(1.15-1.24)	<0.001	1.02	(1.00-1.04)	0.122	0.96	(0.93-0.98)	<0.005
**Pulmonary disease**	1.09	(0.98-1.20)	0.104	1.14	(1.10-1.19)	<0.001	1.16	(1.14-1.18)	<0.001	1.11	(1.08-1.15)	<0.001

**Immunosuppression**	1.22	(1.15-1.30)	<0.001	1.12	(1.08-1.16)	<0.001	1.04	(1.01-1.06)	0.011	1.03	(0.98-1.09)	0.213
**Kidney disease**	1.43	(1.34-1.52)	<0.001	1.32	(1.29-1.36)	<0.001	1.22	(1.20-1.24)	<0.001	1.16	(1.13-1.20)	<0.001
**Obesity**	1.65	(1.61-1.69)	<0.001	1.49	(1.47-1.51)	<0.001	1.34	(1.32-1.36)	<0.001	1.23	(1.18-1.28)	<0.001
**Other comorbidity**	1.28	(1.25-1.31)	<0.001	1.22	(1.20-1.23)	<0.001	1.16	(1.15-1.18)	<0.001	1.13	(1.11-1.15)	<0.001
**Fully vaccinated**	0.96	(0.90-1.03)	0.264	0.99	(0.95-1.04)	0.805	0.96	(0.93-0.98)	<0.005	0.99	(0.96-1.03)	0.742
**Booster**	1.07	(0.95-1.21)	0.275	0.95	(0.88-1.02)	0.132	1.00	(0.96-1.03)	0.808	1.04	(0.99-1.08)	0.101
**Wave 2**	1.13	(1.10-1.16)	<0.001	1.09	(1.07-1.10)	<0.001	0.99	(0.98-1.00)	0.043	0.87	(0.85-0.88)	<0.001
**Wave 3**	1.00	(0.94-1.06)	0.951	1.09	(1.05-1.13)	<0.001	0.97	(0.94-0.99)	<0.005	0.85	(0.82-0.88)	<0.001
**Wave 4**	0.96	(0.87-1.05)	0.349	1.03	(0.97-1.10)	0.308	0.82	(0.79-0.85)	<0.001	0.73	(0.70-0.76)	<0.001
**South**	0.88	(0.86-0.91)	<0.001	0.95	(0.93-0.96)	<0.001	0.96	(0.94-0.97)	<0.001	0.80	(0.78-0.82)	<0.001
**North**	0.77	(0.72-0.83)	<0.001	0.91	(0.88-0.95)	<0.001	0.95	(0.92-0.98)	<0.005	0.93	(0.88-0.98)	0.008
**Northeast**	1.12	(1.08-1.15)	<0.001	1.16	(1.14-1.18)	<0.001	1.20	(1.19-1.22)	<0.001	1.24	(1.21-1.26)	<0.001
**Midwest**	0.99	(0.96-1.03)	0.585	1.04	(1.02-1.06)	<0.001	1.09	(1.08-1.11)	<0.001	1.21	(1.18-1.24)	<0.001
